# (Un)bounded Social Work?—Analysis of Working Conditions in Refugee and Homeless Aid in Relation to Perceived Job Stress and Job Satisfaction

**DOI:** 10.3390/ijerph17020601

**Published:** 2020-01-17

**Authors:** Swantje Robelski, Janika Mette, Tanja Wirth, Niklas Kiepe, Albert Nienhaus, Volker Harth, Stefanie Mache

**Affiliations:** 1 Institute for Occupational and Maritime Medicine (ZfAM), University Medical Centre Hamburg-Eppendorf (UKE), 20459 Hamburg, Germany; swantje.robelski@gmx.de (S.R.); janika.mette@gmx.de (J.M.); niklas.kiepe@bgv.hamburg.de (N.K.); harth@uke.de (V.H.); 2 Competence Centre for Epidemiology and Health Services Research for Healthcare Professionals (CVcare), University Medical Centre Hamburg-Eppendorf (UKE), 20246 Hamburg, Germany; t.wirth@uke.de (T.W.); a.nienhaus@uke.de (A.N.); 3 Department of Occupational Medicine, Hazardous Substances and Public Health, Institution for Statutory Accident Insurance and Prevention in the Health and Welfare Services (BGW), 22089 Hamburg, Germany

**Keywords:** job satisfaction, perceived job stress, resilience, social work, working conditions

## Abstract

Little is known about working conditions of social workers providing help in homeless and refugee aid. Therefore, the present study examined their work-related demands, job and personal resources as well as workplace violence, domain-specific demands, and gender-related differences. Job demands and resources were analyzed with regard to their association with job stress and job satisfaction. Two hundred and fifty-three social workers (69.2% female, 30.8% male) from four federal states in Germany (Berlin, Hamburg, Schleswig-Holstein, and Mecklenburg-Western Pomerania) took part in the cross-sectional quantitative online survey that included validated scales and exploratory items especially developed for the target group. Multiple regression analysis showed that resilience as a personal resource was a significant negative predictor of perceived job stress. Emotional demands were positively related with perceived job stress. Meaning of work and social support were strongly associated with job satisfaction. Language and bureaucratic barriers as well as being affected by clients’ experiences were the domain-specific demands named most often. The study offers insights into the work-related demands and resources and their respective impact on perceived job stress and job satisfaction experienced by social workers in refugee and homeless aid. In order to ensure health and safety for this occupational group, health promotion measures focusing on structural aspects are recommended.

## 1. Introduction

Social workers undertake crucial societal tasks in various areas such as prevention and reduction as well as coping with social problems. Support and counseling offered by social workers targets various client groups and mainly includes person-related services such as consultancy, education, training, and representation [1]. 

There is a growing body of literature on the working conditions experienced by social workers. According to the National Association of Social Workers, social workers have to deal with “increasing barriers to effective practice” [2] (p. 5) such as increases of paperwork, caseloads, and severity of client problems. At the same time, support systems for effective practice were found to be reduced, e.g., by decreasing staff levels and availability of supervision [2]. For social workers, “not being able to get people the help they needed” was described to be the most stressful aspect of their job [3] (p. 336). Lack of resources and organizational restructuring were the main sources of stress identified within the study of Storey and Billingham [4]. Additionally, exposition to conflicting demands as well as the expectation to do things that were not part of the job were the demands named to be occurring most often [3]. Similarly, several studies reported that social workers are exposed to high role conflicts, ambiguity, and low role clarity [5,6,7].

A recurring topic in the context of social work can be seen in the occurrence of violence against social workers. Depending on the sample and measured form of violence, numbers diverge. In this regard, 82% of social workers had experienced to be shouted at or insulted, 46% had received threats of violence, and 23% had already been physically attacked [3]. More than 50% of those who had experienced physical attacks felt very much affected by it [3]. Koritsas and colleagues (2010) described in their results on workplace violence experienced by social workers in Australia that 57% of their sample reported incidents of verbal abuse in the last 12 months. Intimidation was named by 47%, while physical abuse (9%) and sexual assault (1%) were the least common forms of violence [8]. In the Canadian sample obtained by Macdonald and Sirotich (2001), 87.8% of social workers from varying fields of practice reported to have been verbally harassed at least once within their professional career. Threats of physical harm were described by almost two-thirds of study participants [9]. Being subjected to violence has been shown to be related to psychological distress [10]. Work area, gender, as well as client group were found to be associated with different odds of being physically attacked [3]. Hours per week in practice, hours in direct client contact, and age have also been shown to be significant discriminators for differing forms of violence [8]. 

However, there are also several sources of satisfaction described by social workers. They refer to addressing complex problems and achieving accomplishments for their clients [2]. Further sources of satisfaction expressed by social work staff were the feeling of having helped people, progress in a difficult case, challenging work [3], and the team [11]. Aside from resources offered by the job context, resilience has been identified as an important personal resource that was negatively correlated to psychological distress experienced by social workers [12].

With the ongoing refugee influx [13] and a growing number of homeless people in Germany [14], there is a special need for social workers serving these target groups [15]. Only few studies addressed the working conditions in refugee and homeless aid so far, although it can be assumed that there is a number of demands specifically associated with working with refugees and homeless individuals. In this regard, Grimm et al. found in their sample of full-time and voluntary refugee aid workers that low decision latitude, communication problems, and the suffering of people were the most demanding job stressors named [16]. A qualitative study of social workers in refugee and homeless aid was able to show that especially workers’ job content contained emotional and quantitative demands (e.g., high workload, personal traumatic stories of clients), while clients’ appreciation and high control were described as important job resources [17]. 

As a result, health conditions described by social workers often include reports of exhaustion, decreased detachment, and symptoms of burnout or depressive states [17,18,19]. A high incidence of psychological distress and burnout (emotional exhaustion) was found among Spanish social workers [20]. Borho and colleagues reported that about 10% of voluntary and professional refugee aid workers showed at least moderate depressive symptoms [21]. In a longitudinal study, a sample of Polish social workers, a higher level of burnout (t1) was associated with higher levels of secondary traumatic stress (t) [22]. According to Waegemakers-Schiff and Lane, elevated levels of burnout and vicarious traumatization were found in almost a quarter of a sample of workers in homeless aid. Additionally, high rates of traumatic stress were described [23]. There have been reports of higher stress levels for female social workers [4] as well as more severe symptoms of depression for female aid workers [21]. Others found male and female social workers to be more similar than dissimilar [24]. Furthermore, mental disorders are a major cause of sick leave among social workers in Germany, accounting for nearly one fifth of their sick leave days [25]. Similarly, social workers in Finland and Sweden showed a higher risk of work disability with mental diagnoses than other human service professionals such as teachers and psychologists [26]. Gibson et al. described that a high number of social workers scored high in the General Health Questionnaire (GHQ), indicating mild psychiatric morbidity [27]. In the same vein, Balloch and colleagues found high GHQ12 scores for social workers [3]. Aside from health-related impacts, work-related consequences of job stressors can be assumed such as turnover intentions [6] and an impaired quality of service provided [4] or coping strategies including compensatory efforts such as working more intensively or decreased detachment [28].

## 2. Theoretical Background

Using the job demands-resources (JD-R) model as an underlying framework, the mechanisms of social workers’ working conditions, personal and work-related outcomes can be explained. The JD-R assumes that aspects of the job requiring physical or mental effort can be understood in terms of job demands, which are positively related to the impairment of health. Job resources are proposed to be reducing job demands and their adverse effects. Additionally, job resources are supposed to contribute to employees’ motivation and work engagement (motivational process) [29,30]. Since its origins, the JD-R model has been empirically tested and conceptually expanded [31]. In this regard, the job demands-resources model differentiates between job and personal resources which both affect each other as well as the motivational and buffering processes (ibid.). Examples of personal resources that have been studied so far include organizational-based self-esteem, optimism and self-efficacy [32], and psychological wellbeing [33]. 

Stress as an individual reaction to stressors can be understood as a health-related outcome that has been shown to be positively associated with physiological symptomology, depression, anxiety, and fatigue as well as negatively related to life satisfaction [34,35]. Perceived stress as “the degree to which respondents found their lives unpredictable, uncontrollable, and overloading” [34] (p. 387) has therefore been proposed as a state associated with and predictive for future (negative) health-outcomes [34].

Job satisfaction can be seen as an outcome related to the motivational process proposed by the JD-R model. There is sound evidence regarding high correlations between aspects of the psychosocial work environment (e.g., work organization, work content, interpersonal relations) and job satisfaction [36]. Antecedents of job stress and job satisfaction using the JD-R framework have already been surveyed for nurses, showing that “‘work pressure’ and ‘emotional demands’ most consistently related to both job stress and job satisfaction over the review period.” [37] (p. E132). Using the job strain-model, Evans et al. found that mental health social workers with high decision latitude were significantly more satisfied with their jobs than social workers with low decision latitude. Similarly, low job demands were associated with higher job satisfaction than high demands [38]. Coyle et al. (2005) reported in their systematic review that, amongst other factors, workload and being female were factors associated with an increase in stress while feelings of having helped clients was a factor influencing job satisfaction [39].

### Study Aims

**Figure 1:**
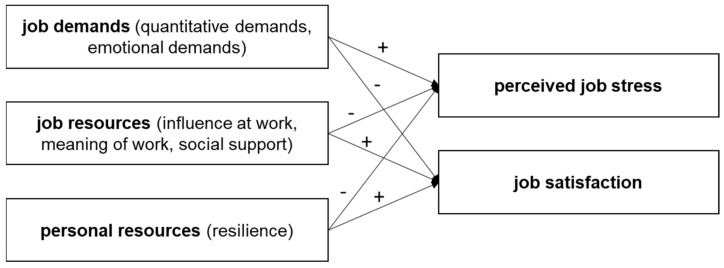
Assumed associations as proposed in hypotheses 1–4.

The aim of the present study was to examine working conditions in terms of specific demands and resources experienced by social workers in refugee and homeless aid and to study potential gender differences. Work-related demands and resources as well as personal resources were investigated regarding their associations with perceived job stress and job satisfaction as can be seen in Figure 1.

**Hypothesis** **1**: Social workers’ perceived job demands, resources, job satisfaction, and job stress differ significantly depending on gender.

- Female social workers perceive higher levels of job demands than male social workers.
- Female social workers perceive higher levels of job resources than male social workers.
- Female workers perceive higher levels of perceived job stress than male social workers.
- Female workers perceive higher levels of job satisfaction than male social workers.

**Hypothesis** **2**: Social workers’ job demands (quantitative demands, emotional demands) are positively associated with perceived job stress

**Hypothesis** **3**: Social workers’ job demands (quantitative demands, emotional demands) are negatively associated with job satisfaction.

**Hypothesis** **4**: Social workers’ job resources (influence at work, meaning of work, and social support) and personal resources (resilience) are negatively associated with perceived job stress.

**Hypothesis** **5**: Social workers job resources (influence at work, meaning of work, and social support) and personal resources (resilience) are positively associated with job satisfaction.

## 3. Materials and Methods 

### 3.1. Study Design and Setting

The study was designed as a cross-sectional study presenting the results of an online survey. Data collection took place between February and May 2019. Social workers were recruited in four federal states located in the north/northeast of Germany (Hamburg, Berlin, Schleswig-Holstein, and Mecklenburg-Western Pomerania).

### 3.2. Participants and Recruitment

Suitable institutions in the refugee and homeless aid were identified by an extensive internet search. Initially, 305 institutions were contacted by email and received leaflets with study information. After some days, the institutions were phoned and asked whether they would like to participate in the survey. While 177 institutions either refused to participate or were not reached, 128 institutions showed interest in participation and forwarded the study information to their employees (participation rate = 41.97%). Inclusion criteria for study participation were employment in an institution in refugee or homeless aid in one of the four federal states, direct contact with refugees and/or homeless individuals, and to be of legal age.

The participating institutions were merely able to provide estimates regarding the number of employees who received the survey link—which were about 1095 employees. Participation in the study was voluntary. Respondents were informed about the study and data confidentiality when entering the survey website and gave written consent subsequently. The website was visited 298 times. Consent was refused 9 times, 21 people did not start the survey, and 15 people did not complete it. In total, 253 online surveys were included in the data analysis (estimated response rate = 23.1%). 

### 3.3. Variables 

#### 3.3.1. Demographic and Workplace Variables

Self-constructed items were used to assess the following variables: gender, age, nationality, federal state, number of inhabitants in place of residence, work area, professional qualification, type of institution, client group, sponsor of institution, professional work experience, working time, type of employment, management responsibility.

#### 3.3.2. Job Demands and Resources

Scales from the Copenhagen Psychosocial Questionnaire (COPSOQ I) were used to assess social workers’ quantitative demands, emotional demands (e.g., “Is your work emotionally demanding?”), social support, influence at work, and meaning of work [36,40]. The items were scored on a 5-point Likert scale (1 = always, 5 = never) and transformed to point values ranging from 0 (minimum) to 100 (maximum) for further analyses. An example item for the scale emotional demands is “Is your work emotionally demanding?” and the item “Do you have a large degree of influence on the decisions concerning your work?” exemplifies the scale on influence at work. The psychometric properties of the COPSOQ I are considered to be good [36,41].

#### 3.3.3. Domain-Specific Job Demands 

**Figure 2:**
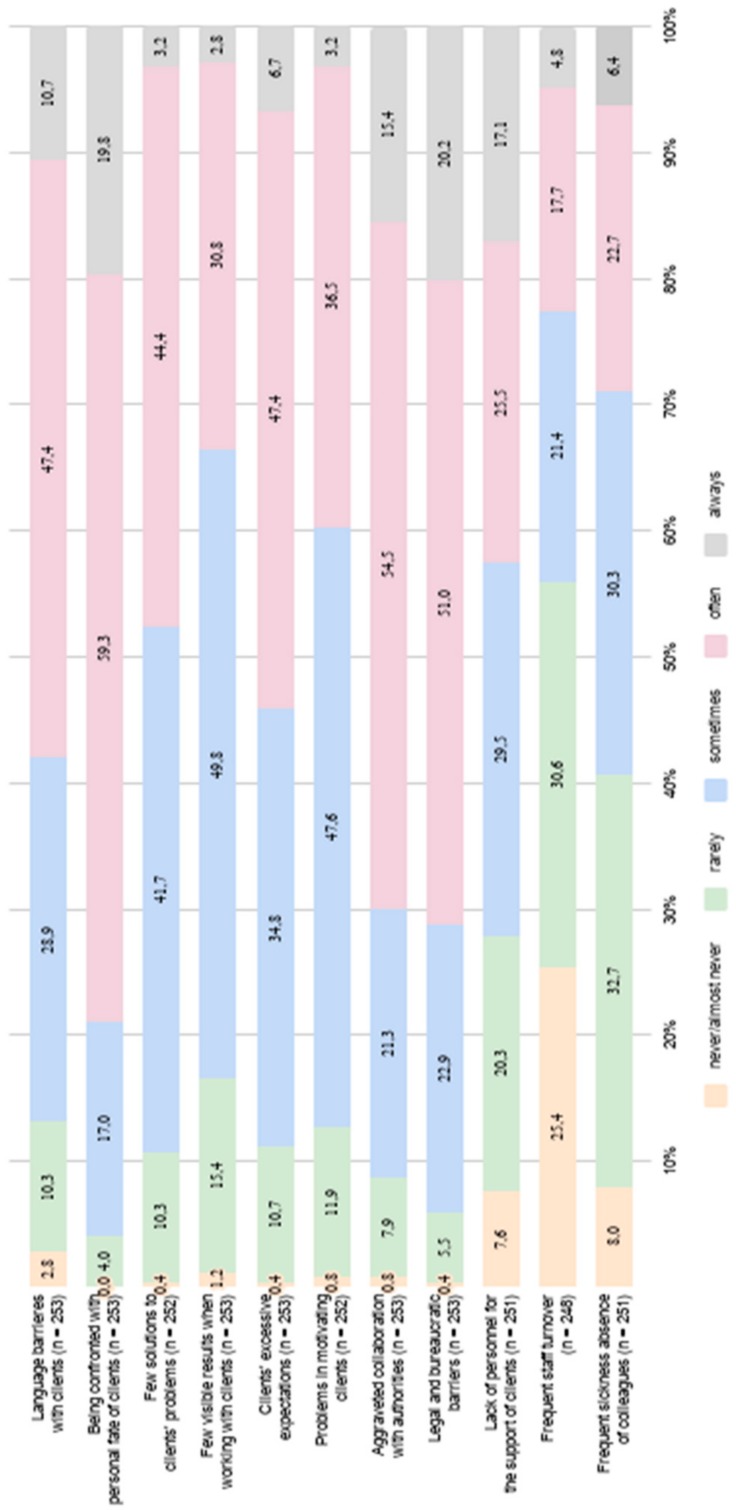
Distribution of domain-specific demands experienced by social workers.

Eleven items on job demands that were assumed to be experienced specifically in the context of refugee and homeless aid were developed and administered based on the results of a previous qualitative study [17]. These items referred to language barriers at work, legal barriers, and confrontation with difficult personal fates of clients. All items can be seen in Figure 2. Scoring was based on a 5-point Likert scale (0 = never, 25 = rarely, 50 = sometimes, 75 = often, 100 = always) that was supposed to resemble the range of the COPSOQ for better comparability.

#### 3.3.4. Workplace Violence

The experience of workplace violence was measured by self-administered measurement items which were also used in a recent German survey on workplace violence in healthcare facilities [42] and are based on the Staff Observation Aggression Scale–Revised (SOAS-R) [43]. Participants had to indicate if they had experienced either physical or verbal aggression of clients in the past twelve months. Respondents confirming the occurrence of physical or verbal aggression were asked to indicate the frequency of such events on a 5-point Likert scale (1 = once a year, 2 = once every three months, 3 = every month, 4 = every week, 5 = daily). 

#### 3.3.5. Perceived Job Stress

The German version of the Perceived Stress Scale (PSS-10) was used to measure the degree to which life in the past month had been experienced as unpredictable, uncontrollable, and overwhelming (e.g., “In the last month, how often have you felt nervous and stressed?”) on a 5-point response scale (0 = never, 1 = almost never, 2 = sometimes, 3 = fairly often, 4 = very often). The PSS-10 total score was obtained by summing up all 10 items. Higher scores indicated a higher level of perceived stress [35]. The PSS was found to be reliable and valid for the assessment of perceived stress [35].

#### 3.3.6. Job Satisfaction

Job satisfaction was measured with a 7-item-scale from COPSOQ III. The verbal response categories are based on a 5-point Likert scale and transformed to point values from 0 (minimum) to 100 (maximum) [44]. The scale includes items such as “Regarding your work in general. How pleased are you with your job as a whole, everything taken into consideration?” The psychometric properties of the COPSOQ were found to be good [36,41]. 

#### 3.3.7. Resilience

A short form of the resilience scale—the RS-13—was used to measure social workers’ resilience. The scale focuses on attributes of the core concept, such as emotional stability, optimism, and vitality [45,46]. The RS-13 consists of 13 items formulated as statements such as “When I make plans, I follow through with them.” Answering is based on a 7-point Likert scale (1 = “I don’t agree”, 7 = “I totally agree”). For scoring, all items are summed up resulting in a range of 13 to 91 with higher scores indicating higher levels of resilience. The psychometric evaluation of the resilience scale showed positive results [46,47].

### 3.4. Statistical Methods

Initial data analysis included plausibility checks and verification for outliers and normality. All necessary data checks for regression modeling were performed (e.g., equality of variances, data homoscedasticity). 

T-tests were conducted to analyze gender differences and Cohen’s d was used as a measure of effect size (small effect—|d| = 0.2, medium-sized effect—|d| = 0.5, large effect - |d| = 0.8) [48]. In order to examine gender differences in categorial variables, chi-square tests were applied. *p*-Values of <0.05 were considered significant. All *p*-values given were two tailed. Spearman’s rho correlation coefficients were used to measure correlations between the variables. In order to test the associations between variables and to determine the predictive power of the job demands and resources examined, two hierarchical regression analyses were conducted. Perceived job stress as well as job satisfaction were used as criterion variables. Predictors were entered into the models based on previous correlation analysis, thus offering the possibility to identify the most predictive variables. Statistical significance level was set at *p* < 0.05. Standardized regressions weights (β) determined the strengths of relations between the variables, whereby β = 0.1 was interpreted as a weak, β = 0.3 as a moderate, and β = 0.5 as a strong association [49]. Additionally, R^2^ was given as a measure of effect size, with scores of 0.02, 0.15, and 0.35 representing small, medium, and large effects [50].

Data were calculated using the IBM^®^ SPSS^®^ Statistics (version 25, IBM, Armonk, NY, USA).

### 3.5. Ethical Considerations

The study was approved by the Medical Ethics Committee of the Hamburg Medical Association, Germany (PV5652).

## 4. Results

### 4.1. Participant Characteristics

Table 1 illustrates socio-demographic characteristics of the participants. In sum, 68.8% were female and 31.3% were male. Most participants were in the age groups of 25–34 years (29.6%) and 35–44 years (27.2%). The majority of the participants were qualified as social workers (62.2%). Almost half of the respondents were employed in the homeless aid (49.6%) and two thirds (75.1%) worked in institutions with an independent sponsor. 

### 4.2. Analysis of Working Conditions in Social Work with Homeless People and Refugees

#### 4.2.1. Demands and Resources

As can be seen in Table 2, all scales administered showed good internal consistency measured by Cronbach’s alpha. Scores are comparable to those attained in different studies [35].

Participants in the present study reported higher emotional than quantitative demands. Among the job resources examined, meaning of work had the highest mean scores.

Table 3 shows the results of the t-tests for gender differences. Significant differences between female and male social workers were found with regard to quantitative (M_female_ 55.95, SD = 17.91, M_male_ = 49.32, SD = 15.95; t(248) = 2.79, *p* < 0.01, d = 0.35) and emotional demands (M_female_ = 68.42, SD = 16.18, M_male_ = 62.07, SD = 15.26; t(248) = 2.92, *p* < 0.01, d = 0.37). Thus, hypothesis 1a can be accepted. With regard to job resources described by female and male social workers, no statistical difference was found. Thus, hypothesis 1b must be rejected. However, men working as social workers in refugee and homeless aid were found to be more resilient than women in the same occupation (t(247) = −2.27, *p* < 0.05, d = 0.29).

Hypothesis 1c assumed that female social workers perceive higher levels of job stress than male social workers, which was confirmed by the statistical analysis (M_female_ = 19.22, SD = 6.67, M_male_ = 16.08, SD = 6.17, t(241) = 3.49, *p* < 0.01, d = 0.45). Job satisfaction was comparable for male and female social workers which leads to the rejection of hypothesis 1d. 

As depicted in Table 4, correlation analysis showed significant positive correlations between quantitative and emotional demands experienced by social workers and perceived job stress (r = 0.31, *p* < 0.000; r = 0.32, *p* < 0.000). Significant negative correlations could be found for perceived job stress and influence at work (r = −0.31, *p* < 0.000), meaning of work (r = −0.17, *p* < 000), social support (r = −0.21, *p* < 0.01), as well as resilience (r = −0.51, *p* < 0.000).

Similarly, correlational analysis revealed significant negative correlations between quantitative demands and job satisfaction (r = −0.17, *p* < 0.01) and emotional demands and job satisfaction (r = −0.17, *p* = 0.007). With regard to job resources, meaning of work showed the strongest correlation with job satisfaction (r = 0.54, *p* < 0.000). Significant positive associations were also found for influence at work (r = 0.40, *p* < 0.000) and social support (r = 49, *p* < 0.000) as well as resilience (r = 0.30, *p* < 0.000).

#### 4.2.2. Domain-Specific Job Demands

As can be seen in Figure 2, further analysis showed that among the domain-specific job demands, being confronted with the personal fate of clients as well as aggravated collaboration with authorities were named to be occurring most frequently. Furthermore, legal and bureaucratic barriers as well as language barriers with clients received high frequency ratings with more than half of the social workers indicating that these domain-specific demands were experienced *often* or *always*.

The comparison of means on these items showed that being confronted with their clients’ personal fates was the demand described with the highest occurrence by social workers (M = 73.72). Bureaucratic barriers (M = 71.25) as well as collaboration with authorities (M = 68.97) also received high frequency ratings. Gender specific differences with regard to the specific demands measured could not be found (all t-tests non-significant), as can also be seen in Table 5. 

#### 4.2.3. Workplace Violence

As data showed, 30.4% of respondents had experienced physical aggression within the last twelve months. Of these, the majority (42.9%) indicated that there was about one incident per year. Some social workers (11.5%) stated that they encountered physical aggression about every three months. With regard to verbal aggression, 75.9% of social workers were able to recall incidents of verbal aggression within the last twelve months, with 27.7% and 19.4% reported to experience verbal aggression once every three months and every month, respectively. For both, the occurrence of physical and verbal aggression, Pearson’s chi-squared test did not indicate significant differences between female and male social workers serving in refugee and homeless aid (physical aggression: χ^2^(1) = 2.46, *p* = 0.12; verbal aggression: χ^2^(1) = 3.53, *p* = 0.06).

### 4.3. Regression Modeling

As can be seen in the first regression model in Table 6, resilience was the strongest predictor for perceived job stress, explaining about 27% of its variance. The more resilient the social workers in the present sample, the less job stress they indicated. In the second model, emotional demands were added as another predictor which resulted in an increment of 0.06 in R^2^ (*p* < 0.000). About the same increase was observed for the addition of quantitative demands in model 3. Therefore, resilience as a personal resource and demands (emotional and quantitative) explained about 38% of variance. In models 4 to 6, predictors that were understood as job resources were added. As can be seen, only model 4, which included influence at work, resulted in a significant change in R^2^ (ΔR^2^ = 0.016, *p* = 0.011). Having more influence at work thus predicted a decrease in perceived job stress, while meaning of work (model 5) and social support (model 6) were non-significant in terms of incremental R^2^-changes. The complete model (6) with all predictors was significant (F_(5, 237)_ = 33.134, *p* <0.000). 

**Figure 3:**
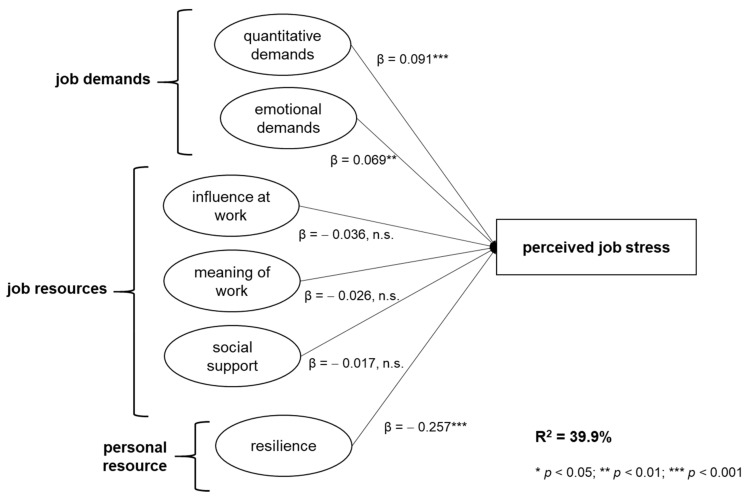
Conceptual model for the outcome perceived job stress with beta-coefficients.

With regard to the proposed hypothesis, hypothesis 2 can be accepted, since both quantitative demands (β = 0.07, *p* < 0.01) and emotional demands (β = 0.09, *p* < 0.001) significantly predicted perceived job stress. Hypothesis 4 can be partially accepted: resilience had a significant negative effect on perceived job stress (β = −0.26, *p* < 0.001), but job resources did not significantly influence job stress. An overview of the model and the coefficients is given in Figure 3.

The regression of job demands, job resources, and resilience as a personal resource on job satisfaction showed significant results, too. In the first model, meaning of work was included as the strongest predictor which explained almost 30% of variance in job satisfaction. In the next step, social support was incorporated, resulting in an increase of ΔR^2^ = 0.14 in explained variance. Model 3 contained influence at work, which was also found to be a significant, positive predictor of job satisfaction. Resilience as a personal resource was added in a fourth model (F_(4, 245)_ = 54.603, *p* < 0.000), revealing a significant regression coefficient of β = 0.18 (st.e. = 0.08, *p* = 0.01). Therefore, hypothesis 5 can be accepted. Models 5 and 6 included emotional and quantitative demands, but while the sixth model was found to be significant (F_(6, 243)_ = 39.909, *p* < 0.000), no significant increase in R^2^ was registered (ΔR^2^ = 0.006, *p* = 0.080). 

**Figure 4:**
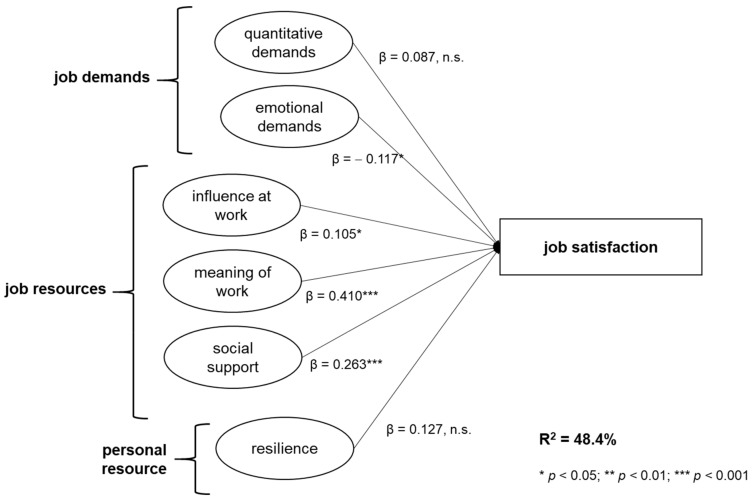
Conceptual model for the outcome job satisfaction with beta coefficients.

Hypothesis 3 can be partially accepted, because emotional demands had a significant negative impact on job satisfaction (β = −0.12, *p* < 0.05), while quantitative demands did not influence job satisfaction (β = 0.09, n.s.). However, model 5 including meaning of work, social support, influence at work, resilience, and emotional demands explained about 48% of variance in job satisfaction. Figure 4 depicts the results for the regression analysis on job satisfaction.

## 5. Discussion

To the best of our knowledge, this is the first study which thoroughly examined the working conditions in German homeless and refugee aid taking into consideration both general psychosocial aspects as well as domain-specific demands experienced by the sample. Significant relations between job demands, job and personal resources, perceived job stress, and job satisfaction were shown.

### 5.1. Descriptive Analysis of Working Conditions and Job-Related Outcomes

In accordance with other studies, high emotional demands were found in this study [51]. At the same time, social workers within the present study expressed a high sense of meaning of work, which has been consistently reported for employees practising a social profession [51]. Domain-specific job demands related to the work with refugees and homeless persons referred to personal fates and hardships experienced by clients, language barriers as well as legal and bureaucratic barriers. This is in line with a recent study showing that burdens experienced by voluntary and professional refugee aid workers include communication problems, aggressive behavior as well as suffering of refugees [21]. Accordingly, a need for psychosocial support and legal information as well as a need to learn how to deal with the demands imposed on them by working in refugee aid were indicated [16,21]. 

Experiences of violence have been quite common for respondents of the present sample. Physical aggression of clients within the last year had been reported by 30% of social workers, while 75% of them were able to recall incidents of verbal aggression for the same period. These numbers are comparable to those reported within other studies of verbal and physical violence experienced by social workers [3,8]. According to Balloch et al., the frequency of violent encounters might be related to job characteristics such as often working alone with clients and not being accompanied by colleagues as well as different client groups [3].

With regard to the outcomes measured in the present study, participants reported more pronounced scores for perceived job stress (M = 18.19, SD = 6.64) than a norm sample (M = 12.57, SD = 6.42) [35]. Job satisfaction was found to be slightly lower than that reported for a broad, national sample of educational and social occupations [52] but comparable to a sample of social workers [51].

### 5.2. Gender Differences Regarding Job Demands, Job Resources, and Job-Related Outcomes

Differences between male and female social workers were found regarding job demands measured with the COPSOQ, while there were no gender differences concerning the perception of domain-specific job demands. However, the latter were based on single item measures which may be associated with smaller explanatory power. In the present study, job resources revealed no differences for men and women. Accessing a large, sector-independent sample showed that women scored higher on the scale for emotional demands, meaning of work, and social support, whereas men indicated more influence at work [52]. In another study, male and female social workers were found to be more similar than dissimilar, although qualitative differences in the use of social support had been proposed [24].

With regard to the outcomes measured, female social workers perceived higher job stress than male social workers, but job satisfaction revealed no differences for men and women in social work with homeless people and refugees. Higher stress levels in female social workers have also been reported by Storey and Billingham [4] and a German norm sample showed that women were scoring higher in the PSS-10 than men, too [35].

Male social workers in our study also scored higher on resilience than female social workers which may be used to explain the differences in the experience of perceived job stress, especially since it was found to be the most predictive factor for perceived job stress in regression modeling.

### 5.3. Associations between Working Conditions and Job-Related Outcomes

Regression analysis confirmed the hypothesized positive association between job demands and perceived job stress. Interestingly, quantitative demands proved to be a stronger predictor for perceived job stress than emotional demands. However, resilience could be identified as a significant protective factor with the highest beta-coefficient. With regard to health promotion measures, resilience trainings might be taken into consideration as a behavioral based approach. Job resources did not reduce perceived job stress as was proposed in our hypothesis and as would be in line with the JD-R.

With regard to job satisfaction, the hypothesized positive relationship with job resources could be confirmed. Among the job resources examined, meaning of work was the strongest predictor. The original validation study of the German COPSOQ showed meaning of work to be an important predictor for job satisfaction, too [41]. Social support was also found to be positively associated with job satisfaction. While a recent qualitative study supported the importance of social support as a job resource [17], the study of Evans was not able to establish a predictive relationship between social support and job satisfaction in a regression analysis, while job demands and decision latitude did [38]. Similarly, our study found that emotional demands were a significant negative predictor for job satisfaction.

### 5.4. Implications for Future Research and Practice

In terms of practical implications, the study results indicate a need for behavioral and structural workplace health promotion measures for social workers in homeless and refugee aid. Structural approaches should specifically target the reduction of employees’ job demands in order to diminish their potentially health-depleting effects. Improvements in the context of work organization are of particular importance, since quantitative demands showed a strong positive association with perceived job stress. In this regard, the reduction of caseloads or higher staffing ratio should be taken into consideration. As social workers showed high emotional demands and suffered from experiencing clients’ personal fates and hardships, they should receive support in the form of regular group or individual supervision and case reviews. The experience of workplace violence was quite frequent in our sample. Therefore, preventive measures and documentation guidelines should be implemented by agencies and authorities. Several recommendations concerning workplace violence in social work have been proposed that also seem to be relevant for the sample of social workers serving in refugee and homeless aid. Suggestions encompass early education on prevention strategies through specific social work training and professional development programmes. Furthermore, on a structural level, guideline and policy development has to be promoted by agencies and authorities, e.g., in the form of standard operation procedures for home visits [8]. Additional aspects of violence prevention refer to office design (e.g., access to doors) as well as continuous training in de-escalation strategies [53]. Behavioral measures such as competence trainings of resilience or self-care can be used to strengthen social workers’ resources on an individual level. In this regard, resilience seems especially important for social workers’ perceived occupational stress. 

Future research should address the subject of job resources more closely and look deeper into why there was no effect on perceived job stress. Which job resources should be taken into consideration? Do they act as moderators between job demands and job stress?

Furthermore, longitudinal studies should be taken into account in order to establish causal relationships between job-related as well as personal predictors and outcomes. In this regard, workplace health intervention studies should be planned and evaluated in order to generate more robust data and ultimately improve working conditions in homeless and refugee aid.

### 5.5. Strengths and Limitations

The present study highlights the importance of understanding working conditions and work-related outcomes in domains such as social work with refugees and homeless people and confirms existing literature on high emotional and quantitative demands. By additionally exploring domain-specific aspects of the job, we were able to get insights into the importance of further job demands. Well-validated instruments were used that have previously shown strong validity and high internal consistency. 

The cross-sectional study design restricts causal conclusions. In addition, survey data was assessed by self-report measures only which may favor bias to the data. The predictive ability of the PSS concerning health-related outcomes is restricted and its results can rather be understood in terms of a broad risk assessment [34]. Representativity across all social workers serving refugees and homeless individuals cannot be assumed.

## 6. Conclusions

Homeless and refugee aid are practice fields of increasing importance among the social work profession and due to the growing number of refugees and homeless people, continuous efforts in creating health-promoting workplaces for social workers are needed. Our study is one of the first to systematically explore working conditions of social workers in homeless and refugee aid. Using hierarchical regression analysis, we were able to highlight how perceived job stress and job satisfaction can be explained by selected job demands and resources (job-related, personal). Results underline the high emotional demands and describe domain-specific job demands such as experiencing personal fates, language and bureaucratic barriers that have been expressed by social workers currently working in the field. Furthermore, job demands were found to be significant predictors of perceived job stress. Therefore, structural as well as individual health promotion measures are strongly recommended in order to avoid a loss spiral of demands and exhaustion [31]. Almost a third of the social workers in the present stated to have experienced physical aggression within the last twelve months. As a study on Iranian social workers showed, being subjected to violence can be seen as a considerable risk factor for burnout [19]. In this regard, trainings and structural measures to protect social workers from mental and physical harm should be implemented.

Job resources had no direct effect on perceived job stress but were positively associated with job satisfaction with meaning of work showing the strongest predictive power. This combination should be observed cautiously with regard to the self-endangerment of social workers [54]. Resilience as a personal resource was found to have a significant protective effect on perceived job stress. Therefore, strengthening social workers’ resources through behavioral health promotion measures could be another task in the course of workplace health interventions. The development and evaluation of such interventions could be one focus of future research in this important field of social work.

## Figures and Tables

**Table 1:** Participant characteristics.

	*n*	%
Gender	250	
Female	173	69.2
Male	77	30.8
Age	253	
≤24 years	1	0.4
25–34 years	74	29.2
35–44 years	69	27.3
45–54 years	52	20.60
≥55 years	57	22.5
Work area	252	
Homeless aid	126	50.0
Refugee aid	98	38.9
Both areas	28	11.1
Professional qualification	258 *	
Social worker	161	62.4
Educator	8	3.1
Social care worker/remedial therapist	4	1.6
Humanities scholar	19	7.4
Law, economics and social sciences	47	18.2
Health-related apprenticeship	8	3.1
Business-related apprenticeship	4	1.6
Other	7	2.7
Work experience in social work	252	
0–3 years	66	26.2
4–10 years	81	32.1
11–20 years	41	16.3
>20 years	64	25.4
Type of institution	245	
Outpatient counselling centre	57	23.3
Day care centre, overnight accommodation	23	9.4
Initial registration centre	3	1.2
Shared accommodation, residential/transition home	100	40.8
Outpatient assisted living	49	20.0
Street social work, street magazine	3	1.2
Emergency shelter	3	1.2
Management, coordination, head office	7	2.9
Sponsor of institution	251	
Independent sponsor (non-profit, charity, church)	191	76.1
Public sponsor	57	22.7
Commercial sponsor (profit-oriented)	3	1.2
Federal state	253	
Berlin	99	39.1
Hamburg	92	36.4
Mecklenburg-Western Pomerania	26	10.3
Schleswig-Holstein	36	14.2

* Multiple choice answer.

**Table 2:** Characteristics of the variables.

Variables		*M*	*SD*	Range	Minimum	Maximum	α
1	Quantitative demands	54.08	17.57	0–100	0	100	0.79
	Female (*n* = 173)	55.95	17.91		13	100	
	Male (*n* = 77)	49,32	15.95		0	88	
2	Emotional demands	66.47	16.17	0–100	8	100	0.77
	Female (*n* = 173)	68.42	16.18		8	100	
	Male (*n* = 77)	62.07	15.26		25	92	
3	Influence at work	53.89	18.89	0–100	0	100	0.74
	Female (*n* = 173)	53.29	19.09		0	100	
	Male (*n* = 76)	55.26	18.79		19	100	
4	Meaning of work	81.39	17.44	0–100	17	100	0.85
	Female (*n* = 173)	81.60	18.53		17	100	
	Male (*n* = 77)	80.63	15.08		42	100	
5	Social support	72.59	20.51	0–100	13	100	0.80
	Female (*n* = 172)	73.00	19.99		17	100	
	Male (*n* = 77)	71.48	21.93		13	100	
6	Job stress	18.19	6.64	0–40	3	37	0.89
	Female (*n* = 167)	19.22	6.67		3	37	
	Male (*n* = 76)	16.08	6.17		3	32	
7	Job satisfaction	64.19	17.00	1–100	14	100	0.82
	Female (*n* = 173)	64.17	17.44		14	100	
	Male (*n* = 77)	64.49	16.30		21	96	
8	Resilience	68.76	11.09	13–91	32	90	0.88
	Female (*n* = 172)	67.56	11.58		32	90	
	Male (*n* = 77)	70.99	9.49		52	90	

**Table 3:** T-tests of working conditions for male and female social worker.

Variables		T	Df	Mean diff.	SED	Lower Confidence	Upper Confidence
1	Quantitative demands	2.79 **	248	6.63	2.38	1.95	11.30
2	Emotional demands	2.92 **	248	6.36	2.18	2.07	10.65
3	Influence at work	−0.75	247	−1.98	2.62	−7.13	3.18
4	Meaning of work	0.44	177.13	0.97	2.22	−3.41	5.36
5	Social support	0.54	247	1.52	2.83	−4.05	7.08
6	Resilience	−2.27 *	247	3.42	1.51	−6.39	−0.46
7	Perceived job stress	3.49 **	241	3.14	0.90	1.37	4.92
8	Job satisfaction	−0.14	248	2.28	2.34	−4.94	4.29

*Note*: *t*-test: * *p* < 0.05; ** *p* < 0.01.

**Table 4:** Spearman correlation coefficients for all variables.

Variables		1	2	3	4	5	6	7	8
1	Quantitative demands	-							
2	Emotional demands	0.35 ***	-						
3	Influence at work	−0.14	−0.05	-					
4	Meaning of work	0.10	0.07	0.40 ***	-				
5	Social support	−0.23 ***	−0.15 *	0.24 ***	0.23 ***	-			
6	Resilience	−0.05	−0.22 **	0.28 ***	0.30 ***	0.13 *	-		
7	Perceived job stress	0.31 ***	0.32 ***	−0.31 ***	−0.17 **	−0.21 **	−0.51 ***	-	
8	Job satisfaction	−0.17 **	−0.17 **	0.40 ***	0.54 ***	0.49 ***	0.30 ***	−0.36 ***	-

*Note:* Spearman correlation coefficient: * *p* < 0.05; ** *p* < 0.01; *** *p* < 0.001.

**Table 5:** T-tests on domain-specific demands for male and female social workers.

Variables		M	SD	T	df
1	Language barriers with clients	63.24	22.88	0.74	248
	Female (*n* = 173)	64.02	23.70		
	Male (*n* = 77)	61.69	21.30		
2	Personal fate of clients	73.72	18.12	1.26	248
	Female (*n* = 173)	74.57	18.58		
	Male (*n* = 77)	71.43	17.07		
3	Few solutions to clients’ problems	59.92	18.29	−0.13	247
	Female (*n* = 172)	59.74	18.43		
	Male (*n* = 77)	60.06	18.25		
4	Few visible results when working with clients	54.64	19.18	0.92	248
	Female (*n* = 173)	55.35	18.98		
	Male (*n* = 77)	52.92	19.86		
5	Clients’ excessive expectations	62.35	19.75	0.72	248
	Female (*n* = 173)	63.01	20.81		
	Male (*n* = 77)	61.04	17.44		
6	Problems in the motivation of clients	57.34	18.69	−1.30	247
	Female (*n* = 172)	56.40	17.81		
	Male (*n* = 77)	59.74	20.74		
7	Aggravated collaboration with authorities	68.97	20.91	1.93	248
	Female (*n* = 173)	70.66	18.95		
	Male (*n* = 77)	64.61	24.45		
8	Legal and bureaucratic barriers	71.25	20.43	1.40	248
	Female (*n* = 173)	72.40	19.07		
	Male (*n* = 77)	68.18	23.17		
9	Lack of personnel for the support of clients	56.08	29.50	0.17	246
	Female (*n* = 172)	55.96	29.32		
	Male (*n* = 76)	55.26	30.09		
10	Frequent staff turnover	36.49	29.66	0.6	243
	Female (*n* = 171)	36.40	29.73		
	Male (*n* = 74)	36.15	30.17		
11	Frequent sickness absence of colleagues	46.71	26.39	−1.06	246
	Female (*n* = 172)	45.49	25.89		
	Male (*n* = 76)	49.34	27.68		

*Note*: t-test: * *p* < 0.05; ** *p* < 0.01; *** *p* < 0.001.

**Table 6:** Regression analysis on perceived job stress and job satisfaction.

Job Stress	1		2		3		4		5		6	
	β	Std. E.	β	Std. E.	β	Std. E.	β	Std. E.	β	Std. E.	β	Std. E.
Resilience	−0.32 ***	0.03	−0.29***	0.03	−0.29 ***	0.03	−0.27 ***	0.03	−0.26 ***	0.03	−0.26 ***	0.03
Emotional demands			0.10***	0.02	0.06 **	0.02	0.07 **	0.01	0.07 **	0.02	0.07 **	0.02
Quantitative demands					0.10 ***	0.02	0.09 ***	0.02	0.09 ***	0.02	0.09 ***	0.02
Influence at work							−0.05 *	0.02	−0.04	0.02	−0.04	0.02
Meaning of work									−0.03	0.02	−0.03	0.02
Social support											−0.02	0.02
n	243		243		243		243		243		243	
R²	0.28		0.33		0.39		0.40		0.41		0.41	
adj. R²	0.27		0.33		0.38		0.40		0.40		0.40	
Job satisfaction	1		2		3		4		5		6	
	β	Std. E.	β	Std. E.	β	Std. E.	β	Std. E.	β	Std. E.	β	Std. E.
Meaning of work	0.54 ***	4.31	0.46 ***	0.05	0.40 ***	0.05	0.37 ***	0.05	0.40 ***	0.05	0.41 ***	0.05
Social support			0.32 ***	0.04	0.29 ***	0.04	0.29 ***	0.04	0.28 ***	0.04	0.26 ***	0.04
Influence at work					0.14 **	0.05	0.12 *	0.05	0.12 *	0.05	0.11 *	0.05
Resilience							0.18 *	0.08	0.12	0.08	0.13	0.08
Emotional demands									−0.15 **	0.05	−0.12 *	0.05
Quantitative demands											−0.09	0.05
*n*	250		250		250		250		250		250	
R²	0.30		0.44		0.46		0.47		0.49		0.50	
adj. R²	0.30		0.44		0.45		0.46		0.48		0.48	

*Note.* * *p* < 0.05; ** *p* < 0.01; *** *p* < 0.001.
